# Case report: An intraretinal macrocyst with crystalline content and retinal detachment

**DOI:** 10.3389/fmed.2022.1051181

**Published:** 2022-12-15

**Authors:** Chang Cai, Jialin Zhou, Qiuyu Wang, Weihou Li, Danning Liu

**Affiliations:** Department of Ophthalmology, Second Affiliated Hospital of Chongqing Medical University, Chongqing, China

**Keywords:** B-scan ultrasonography, case report, crystalline contents, intraretinal macrocyst, vitrectomy

## Abstract

**Introduction:**

An intraretinal macrocyst is a cavity located in the outer plexiform layer of the retina. It is commonly filled with liquid or blood. To date, few case reports of intraretinal macrocysts with crystalline content and retinal detachment have been published.

**Case presentation:**

A 44-year-old woman with no history of other diseases complained of decreased vision in her right eye that had persisted for 20 days. The best corrected visual acuity of the right eye was hand motion. Comprehensive ophthalmic examinations were performed, including a vision test, slit lamp fundus examination, ocular B-scan ultrasound, and orbital magnetic resonance imaging. We performed vitrectomy and retinotomy to sufficiently remove the macrocyst and relieve retinal traction. We then reattached the retina and filled it with silicone oil. During the surgery, we found that the cyst had crystalline content, which has not been previously reported, to the best of our knowledge. Finally, the pathological results confirmed a final diagnosis of intraretinal macrocyst. Six months later, we performed a second operation to remove the silicone oil and implant an intraocular lens. After both surgeries, the best corrected visual acuity of the patient's right eye was restored to 20/200, and the retina had repositioned.

**Conclusion:**

Intraretinal macrocysts are very rare. Both orbital magnetic resonance imaging and ocular B-scan ultrasound are essential for their diagnosis. Our results indicated that vitrectomy was the best way to remove the cyst and reattach the retina.

## Introduction

An intraretinal macrocyst is usually located in the outer plexiform layer of the retina, separating its inner and outer nuclear layers (1). Most researchers believe that retinal cysts are secondary to long-standing retinal detachment (2). This case report describes a rare intraretinal macrocyst with crystalline content and long-standing retinal detachment.

## Case presentation

A 44-year-old woman saw drifting dark shadows and experienced blurred vision in her right eye for 20 days. The symptoms worsened after 3 days with an upper visual field defect. She had no history of trauma or other diseases. The best corrected visual acuity of the right eye was hand motion. A slit-lamp biomicroscope showed a posterior subcapsular cataract of the right eye. The fundus was blurred. The left eye was generally normal. Although the patient experienced a short course of the disease, we found a significant exotropia of −15° in the right eye with no fixation ability. Intraocular pressure was normal in both eyes; 11.7 mmHg in the right eye and 12.5 mmHg in the left.

**Figure 1:**
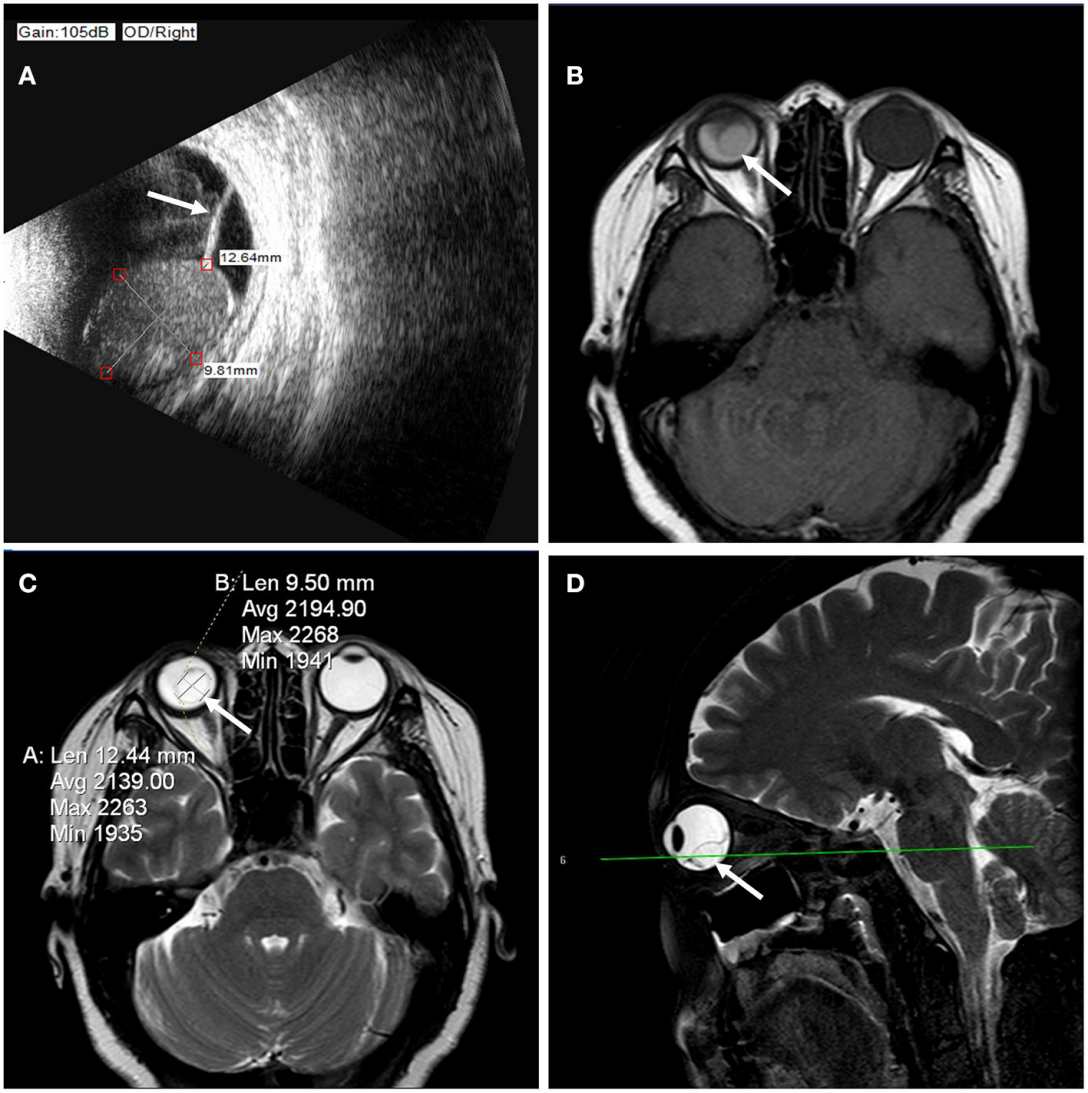
**(A)** B-ultrasound images showed an intraretinal macrocyst and a detached retina (arrow). The size of the intraretinal macrocyst was approximately 12.64 × 10.42 mm. **(B)** Orbital MRI revealed a moderate-enhancing mass (arrow) in T1 images in the vitreous cavity of the right eye. **(C)** Orbital MRI revealed a hyper-enhancing mass (arrow) in T2 images in the vitreous cavity of the right eye. The hyper-enhancing mass was approximately 12.44 × 9.50 mm. **(D)** The lesion (arrow) was located in the posterior lower part of the vitreous cavity on sagittal scans.

A B-scan ultrasound confirmed the presence of a 12.64 x 10.42-mm smooth cystic mass with moderate internal echo and a hyperechoic cyst wall in the vitreous cavity of the right eye. A shallow, straight hyperechoic band was continuous with the mass wall and attached to the eyeball wall at the other end (Figure 1A). Orbital magnetic resonance imaging (MRI) also revealed a moderate-enhancing mass in T1 images (Figure 1B) and a hyper-enhancing mass in T2 images (Figure 1C) in the vitreous cavity of the right eye. They both had the same enhancement as the abnormal vitreous body of the right eye, accompanied by a hypo-enhancing smooth wall. In addition, the presence of the same hypo-enhancing band connected the mass to the wall of the eye (Figure 1D). Before surgery, a diagnosis of an intraretinal hemorrhagic cyst with rhegmatogenous retinal detachment (RRD) was established.

**Figure 2:**
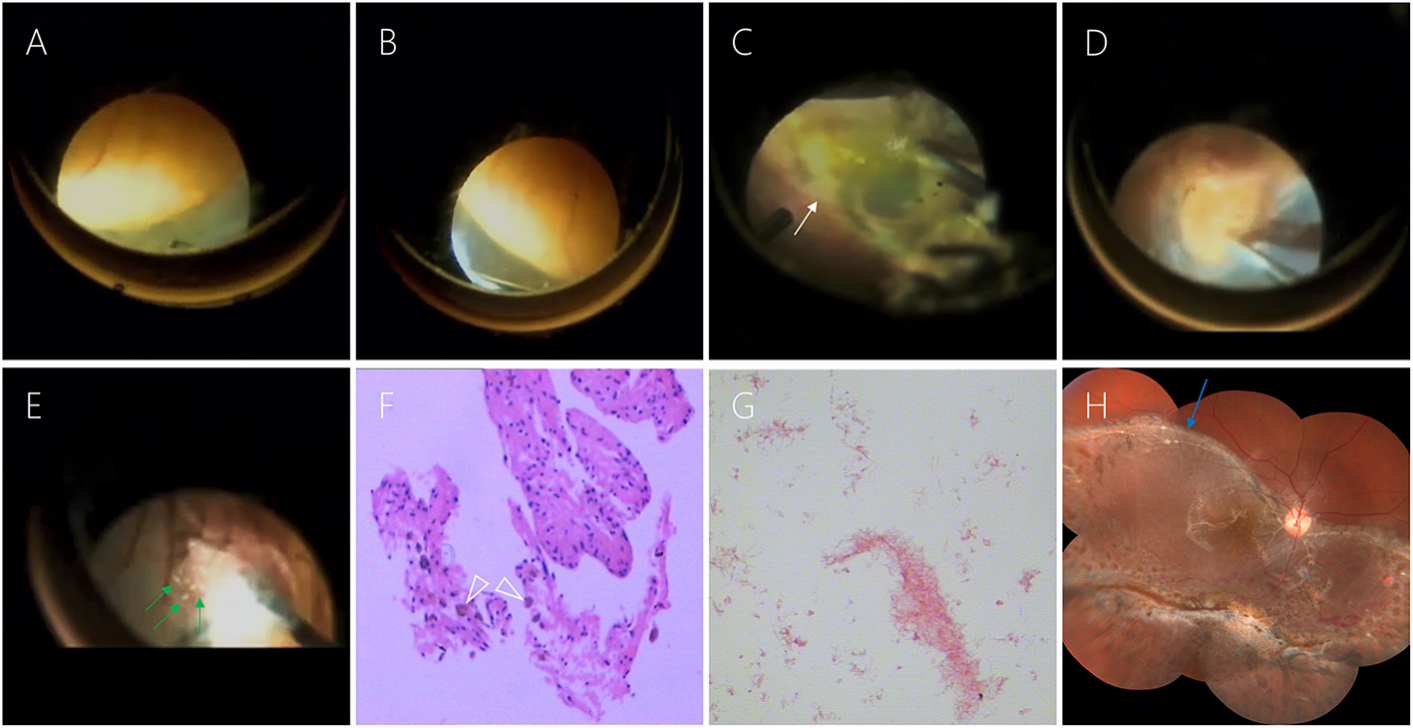
**(A, B)** The giant cyst was located in the inferior peripheral area from 4 to 8 o'clock. **(C)** There was a clear boundary of epithelial atrophy (white arrow) between the detached retina and the normal one. **(D)** After removing the upper retina, we can see the apparent cyst wall. **(E)** Colored crystalline fluid (green arrow) flowed from the cyst when the retina and the wall were incised. **(F)** The pathological results of the cyst wall revealed a small amount of retinal tissue and pigmentation (white triangle). **(G)** The abscission cytology test of the crystalline liquid excluded the presence of nucleated cells. **(H)** Six months after the first surgery, the retina had been repositioned. The boundary between the normal retina and the detached retina that appeared earlier could be visualized (blue arrow).

The patient agreed to undergo a 23-G vitrectomy to remove the large cyst and attach the retina. The giant cyst was located in the inferior peripheral area from 4 to 8 o'clock (Figures 2A,B). Extensive retinal detachment occurred at the posterior edge of the cyst in the 3–9 o'clock direction with an extension to the macula and subretinal cord-like proliferation. There was a clear boundary of epithelial atrophy between the detached retina and the normal one (Figure 2C). However, we did not find any retinal breaks. Instead, we identified an apparent cyst wall (Figure 2D). Colored crystalline fluid, instead of blood, flowed from the cyst when the retina and the wall were incised (Figure 2E). Although we only cut a small hole in the retinal cyst, the crystalline contents flowed into the vitreous cavity quickly. Unfortunately, we were unable to obtain the crystalline material to analyze its composition. We performed a 180° retinotomy near the serrated edge, removed the cyst and subretinal proliferation, and fully released the retinal traction. We then reattached the retina and filled it with silicone oil. The pathological results of the cyst wall revealed a small amount of retinal tissue and pigmentation (Figure 2F). The abscission cytology test of the crystalline liquid excluded the presence of nucleated cells (Figure 2G). A second surgery was scheduled 6 months later to remove the silicone oil. At the same time, we performed phacoemulsification and implanted an intraocular lens. After the second surgery, the best corrected visual acuity of the patient's right eye was restored to 20/200, and the retina had repositioned. The boundary between the normal retina and the detached retina that appeared earlier could be visualized (Figure 2H).

## Discussion

This case report presented an intraretinal macrocyst with characteristic multimodal images before vitrectomy. The pathological results after surgery allowed for a differential diagnosis. In addition, the surgery confirmed the appearance of a rare colored crystalline content.

According to Hagler and North (3), an intraretinal macrocyst is defined as an isolated retinal cyst of medium-size associated with retinal detachment. Many researchers have subsequently highlighted the relationship between intraretinal cysts and long-standing retinal detachment (4). Retinal cysts are generally believed to be secondary to RRD. In addition, the large cysts further prevent retinal break closure and retinal reattachment (2, 5). The pathogenesis of retinal cysts is unclear, although it is thought to be photoreceptor atrophy secondary to choroidal ischemia and degenerative cystic changes in the outer plexiform layer of the retina (4). Some retinal cysts have been reported to be associated with Coats' disease and toxoplasmosis, and can even exist idiopathically (6–8). In the present case, we found an apparent retinal detachment linked to the cyst.

However, of note, there were no retinal holes, and associated primary retinal or choroidal disease was detected during surgery. There was no evidence to support the cause of cyst formation other than retinal detachment. Therefore, what causes cyst formation and retinal detachment? We propose two hypotheses. Firstly, we hypothesize that a micro-hole in the peripheral retina leads to asymptomatic retinal detachment. The micro-hole may then be resealed during RRD. However, RRD can lead to functional changes in the retina, modifying blood flow and metabolic events (9). As a result, cysts are formed and gradually become more prominent. The larger the cyst grows, the more difficult it is to reattach the retina. Secondly, we hypothesize that initial retinal detachment is actually retinoschisis with a large cyst. Gradually the inflammation disappeared and we only saw huge cysts and shortened retinal detachment. Keith and Sen reported similar cases of retinoschisis and large retinal cysts (1, 10).

Retinal macrocysts are commonly filled with fluid and occasionally blood. Most of the hemorrhagic contents are formed by rupture of the retinal vessels during retinal degeneration (11). However, to the best of our knowledge, this is the first report of a macrocyst with colored crystalline content. The colored crystalline content was confirmed intraoperatively and showed distinct moderate echogenicity in ultrasound and orbital MRI images. Many studies have demonstrated that in RRD with micro-holes, the subretinal fluid is usually cloudy and viscous (11, 12). The fluid in the cyst cavity is not very different from the subretinal fluid (13). In the present case, we also confirmed unclear fundus findings due to vitreous clouding. The same enhancement was seen in the vitreous cavity and the macrocyst on B-scan ultrasound and MRI images. However, during the surgery, yellow fluid, instead of crystalline fluid, was found in the vitreous cavity. The duration of retinal detachment and cyst formation may differ, resulting in different fluid absorption periods. In short, they both can be used as evidence of the long disease course.

Furthermore, MRI is more helpful in distinguishing the cyst from the wall of the eye, which can be used as a differential diagnosis for retinal and choroidal tumors, such as retinoblastoma, choroidal melanoma, and solitary choroidal hemangioma, especially when the mass contents are hyper-enhancing (4). However, the final diagnosis in the present case was confirmed by pathological and exfoliative cytology tests. Although the pathological findings and the late clinical manifestations didn't provide any evidence of cysticercosis or other parasitic entities, the initial inflammatory causes are still difficult to exclude.

We believe that the key to successful surgery is the removal of the macrocyst that obstructs retinal release, in support of Marcus and Kumar, who recommend the surgical removal of the cyst if it prevents retina reconnection (14, 15). We attempted at first to cut a small hole in the periphery of the cyst to remove the intraretinal fluid inside the cyst. Then we found another problem. The detached retina below the cyst shortened and was hard to reattach. Therefore, we had to make a retinotomy in the peripheral retina to loosen and flatten the retina. We tried to preserve the retina containing the cyst wall to avoid giant tears and choroidal exposure. In the case of small cysts, some are of the opinion that they do not require specific treatment. It can take several years for collapse to occur spontaneously after successful retinal reattachment (11). The critical premise of all of these views is that the retina can be reattached. The inner wall of the cyst is composed of Miller's fibers and the nucleus in the inner nuclear layer (1). In the present case, the pathological findings revealed that the cyst wall comprised retinal tissue. Liu et al. also pointed out that retinal cysts do not have an epithelial lining, only fluid-filled cavities (12). Therefore, draining the cyst without completely removing the cyst wall can lead to fluid reaccumulating. In addition, we emphasize that sufficient relief of retinal traction by retinotomy is helpful for retinal reattachment in RRD. Hocaoglu et al. (13) reported a 94% primary surgical success rate after retinotomy/retinectomy for RRD with proliferative vitreoretinopathy.

## Conclusion

Orbital MRI and ocular B-scan ultrasound were both essential for the diagnosis and differential diagnosis of intraretinal macrocysts through characteristic images before surgery. The pathological results confirmed that the cyst was derived from retinal tissue. Vitrectomy with retinotomy to remove the macrocyst and sufficiently relieve retinal traction was effective in this case.

## Data availability statement

The original contributions presented in the study are included in the article/supplementary material, further inquiries can be directed to the corresponding author.

## Ethics statement

The studies involving human participants were reviewed and approved by the Ethics Committees of the Second Affiliated Hospital of Chongqing Medical University. The patients/participants provided their written informed consent to participate in this study. Written informed consent was obtained from the individual(s) for the publication of any potentially identifiable images or data included in this article.

## Author contributions

DL provided this case and offered guidance. DL and CC performed the data analysis and wrote the manuscript. JZ, QW, and WL contributed to discussion. All authors contributed to the article and approved the submitted version.

## References

[1] Keith CG. Retinal cysts and retinoschisis. Br J Ophthalmol. (1966) 50:617–28. 10.1136/bjo.50.11.617 5332827 PMC506288

[2] Verdaguer P Nadal J. Intraretinal cyst secondary to longstanding retinal detachment. Eur J Ophthalmol. (2012) 22:506–8. 10.5301/ejo.5000034 21928257

[3] Hagler WS North AW. Intraretinal macrocysts and retinal detachment. Trans Am Acad Ophthalmol Otolaryngol. (1967) 71:442–54. 6032914

[4] Naik AU Rishi P Prakash VJ Rishi E. Retinal detachment with hemorrhagic intraretinal macrocyst clinically presenting as pseudo-choroidal melanoma. Oman J Ophthalmol. (2019) 12:62–4. 10.4103/ojo.OJO_136_2018 30787541 PMC6380148

[5] Serna-Ojeda JC Serna-Ojeda JC Pinkus-Herrera CD Moreno-Londono MV Rodriguez-Loaiza JL Gonzalez-Gonzalez MC. Clinical and echographic long-term follow-up of a retinal macrocyst: a case report. Case Rep Ophthalmol. (2014) 5:168–71. 10.1159/000363759 25028580 PMC4086044

[6] Karimi S Nikkhah H Fekri S. Ocular toxoplasmosis presenting as subretinal macrocyst. J Ophthalmic Vis Res. (2019) 14:223–5. 10.4103/jovr.jovr_210_16 31114661 PMC6504721

[7] Chen CY Semenova E Cohen BZ Finger PT. Idiopathic giant retinal cyst. Ophthalmic Surg Lasers Imaging Retina. (2014) 45:251–2. 10.3928/23258160-20140501-04 24840531

[8] Munira Y Zunaina E Azhany Y. Resolution of exudative retinal detachment and regression of retinal macrocyst post-laser in Coats disease. Int Med Case Rep J. (2013) 6:37–9. 10.2147/IMCRJ.S47769 23966803 PMC3743637

[9] Labriola LT Brant AM Eller AW. Chronic retinal detachment with secondary retinal macrocyst and peripheral neovascularization. Semin Ophthalmol. (2009) 24:2–4. 10.1080/08820530802508561 19241283

[10] Sen P Mishra S. Surgical management of a large retinal cyst in X-linked retinoschisis with internal drainage: Report of an unusual case. Indian J Ophthalmol. (2020) 68:2294–6. 10.4103/ijo.IJO_2336_19 32971698 PMC7727930

[11] Rishi P Rishi E Sen PR Sharma T. Hemorrhagic intraretinal macrocyst: Differential diagnoses and report of an unusual case. Oman J Ophthalmol. (2011) 4:28–31. 10.4103/0974-620X.77660 21713238 PMC3110444

[12] Liu TYA Alvin Liu TY Vizcaino MA Eberhart CG Sachdeva MM. Association of macular and peripheral retinal macro-pseudocysts with chronic retinal detachment. JAMA Ophthalmol. (2018) 136:956–8. 10.1001/jamaophthalmol.2018.2189 29902288 PMC6492548

[13] Hocaoglu M Karacorlu M Ersoz MG Muslubas IS Arf S. Retinotomy and retinectomy for anterior inferior proliferative vitreoretinopathy: can visual outcome be improved. Eur J Ophthalmol. (2022) 32:1136–42. 10.1177/11206721211012848 33887980

[14] Marcus DF Aaberg TM. Intraretinal macrocysts in retinal detachment. Arch Ophthalmol. (1979) 97:1273–5. 10.1001/archopht.1979.01020020015003 454261

[15] Kumar V Vivek K Chandra P Kumar A. Ultrawide field imaging of multiple intraretinal cysts in old rhegmatogenous retinal detachment. Oman J Ophthalmol. (2016) 9:191–2. 10.4103/0974-620X.192309 27843242 PMC5084510

